# Supplementary material for: Cytokinin transfer by a free-living mirid to Nicotiana attenuata recapitulates a strategy of endophytic insects
Source: eLife. 2018 Jul 17;7:e36268. doi: 10.7554/eLife.36268 (PMC6059766; doi:10.7554/eLife.36268)
Supplement: Supplementary file 1. [file elife-36268-supp1.docx]

Supplementary File 1: Calculations of the minimum amount of IP transferred by a single mirid in clip-cage experiment and estimation of the number of feeding mirids required to transfer the measured amount of IP in the whole-plant experiment

|  | | | | |
| --- | --- | --- | --- | --- |
| After 120 h continuous feeding | **Clip cage experiment** | | **Whole-plant experiment** | |
|  | **Transferred**  **[^15^N_5_]-IP/IPR:**  **fmol / g FM leaf** | **fmol IP/mirid** | **Transferred**  **[^14^N_5_]-IP/IPR: fmol / g FM leaf** | **estimated number of mirids on one leaf:** |
|  |  |  |  |  |
| IP | 2.35 | 0.117 | 15.6 | 133 |
| IPR | 34.9 | 1.742 | 135.4 | 78 |

Clip cage experiment: 20 ^15^N labeled mirids feeding on single leaves of ^14^N-grown plants for 5 days.

Whole-plant experiment: ^15^N-grown plants exposed to the feeding of an unknown number of ^14^N labeled mirids.

FM: fresh mass.
