# Supplementary material for: Cytokinin transfer by a free-living mirid to Nicotiana attenuata recapitulates a strategy of endophytic insects
Source: eLife. 2018 Jul 17;7:e36268. doi: 10.7554/eLife.36268 (PMC6059766; doi:10.7554/eLife.36268)
Supplement: Supplementary file 2. [file elife-36268-supp2.docx]

Supplementary File 2: Sequences of primers used for real-time qPCR

| **Gene** | **Forward primer** | **Reverse primer** |
| --- | --- | --- |
| *NaActin* | 5' GGTCGTACCACCGGTATTGTG 3' | 5' GTCAAGACGGAGAATGGCATG 3' |
| *NaCKX5* | 5' TTGTCGGCTTATTGTAACCGTCG 3' | 5' GTTAAGAACTGCCATCGGCTC 3' |
| *NaRRA5* | 5'AGATGAGTTGCATGTTCTTGCTGT3' | 5' TCAATCCCCACAGAGGTCTTCT 3' |
| *NaZOG2* | 5' GTCATGCAAGTCAATTTAAGAGCTC 3' | 5' AGGAAATTTGGGAAGAAGGTGTAAG 3' |
| *NaLOG4* | 5' CTCAGCTCACAAAGTCTTCACG 3' | 5' CCATTAAGCCAACACTTCCACC 3' |
| *NaIPT5* | 5' TCAGCCACTTATTAATTTCCGAGAG 3' | 5' TTGGCTAGATCAATGGATAGTCTAG 3' |
