# Supplementary material for: Cytokinin transfer by a free-living mirid to Nicotiana attenuata recapitulates a strategy of endophytic insects
Source: eLife. 2018 Jul 17;7:e36268. doi: 10.7554/eLife.36268 (PMC6059766; doi:10.7554/eLife.36268)
Supplement: Supplementary file 3. [file elife-36268-supp3.docx]

Supplementary File 3. Multi-reaction monitoring settings for the quantification of [^14^N_5_]-, [^15^N_5_]- and deuterated cytokinins in positive ionization mode.

Analyte RT [min] Q1 [m/z] → Q3 [m/z]^a,b^ CE [V]^a^ Standard^c^

[^15^N_5_]-IP 5.12 (+)209.10 → 141.00 -14 D_6_-IP

[^15^N_5_]-IPR 6.14 (+)341.10 → 209.30 -12 D_6_-IPR

(+)341.10 → 141.00 -28

*c*Z 2.53 (+)220.20 → 136.30 -16 D_5_-*t*Z

(+)220.20 → 148.30 -16

*c*ZR 4.45 (+)352.20 → 220.30 -16 D_5_-*t*ZR

(+)352.20 → 136.00 -25

IP 5.18 (+)204.10 → 136.00 -14 D_6_-IP

IPR 6.10 (+)336.10 → 204.30 -12 D_6_-IPR

(+)336.10 → 136.50 -28

*t*Z 2.25 (+)220.20 → 136.30 -16 D_5_-*t*Z

(+)220.20 → 148.30 -16

*t*ZR 4.04 (+)352.20 → 220.30 -16 D_5_-*t*ZR

(+)352.20 → 136.00 -25

D_6_-IP 5.11 (+)210.10 → 137.00 -14

D_6_-IPR 6.04 (+)342.00 → 210.00 -12

(+)342.00 → 136.50 -28

D_5_-*t*Z 2.22 (+)225.20 → 136.60 -16

D_5_-*t*ZR 3.98 (+)357.20 → 225.50 -16

RT: retention time

CE: collision energy

^a^ Qualifiers are depicted in grey

^b^ Resolution: Q1: 0.7, Q3: 2
